# Case Report: Endoscope-assisted single-incision double-channel mini-open hemilaminectomy for the treatment of acute thoracolumbar intervertebral disc disease in 11 dogs

**DOI:** 10.3389/fvets.2025.1543611

**Published:** 2025-04-09

**Authors:** Hao Shi, Qi Wang, Zhurui Shao, Haojie Xu, Yufei Yang, Yiwen Zhang, Ruizi Ren, Jieen Weng

**Affiliations:** ^1^The Clinical Department, College of Veterinary Medicine, China Agricultural University, Beijing, China; ^2^China Agricultural University Veterinary Teaching Hospital, Beijing, China; ^3^Babara Veterinary Hospital, Shanghai, China

**Keywords:** dog, intervertebral disc disease, minimally invasive surgery, neurosurgery, mini-open hemilaminectomy

## Abstract

This study aims to explore the feasibility and efficacy of an endoscopic-assisted mini-open hemilaminectomy technique for spinal cord decompression in thoracolumbar intervertebral disc extrusion. A total of 11 dogs with acute thoracolumbar intervertebral disc disease were included in the study, preoperative magnetic resonance imaging (MRI) and computed tomography (CT) were used for precise localization. The surgery was performed using a lateral approach with a skin incision approximately 2 cm in length for a minimally invasive hemilaminectomy of the thoracolumbar spine. After separating the epaxial musculature below the articular process and exposing the tendon attachment of the accessory process, the endoscope and surgical instruments were placed. A nerve hook and nucleus pulposus forceps were used to remove the thoracolumbar intervertebral disc extrusions and relieve spinal cord compression. Postoperative MRI or CT confirmed complete removal of the disc extrusions with no significant complications observed, and all dogs exhibited normal gait and neurological examination results. This technique demonstrated advantages such as easy handling minimal incision, precise localization, and reduced iatrogenic damage, resulting in good postoperative recovery. This case series demonstrates that the endoscopic-assisted mini-open hemilaminectomy technique can safely be implemented to decompress the spinal cord in dogs. This novel technique adds onto the current growing surgical options for minimally invasive spinal surgery in veterinary neurosurgery.

## 1 Introduction

Canine intervertebral disc disease (IVDD) is one of the most common spinal disorders in dogs, with a peak incidence typically occurring between 3 and 6 years of age. It primarily affects chondrodystrophic breeds, with a particularly high prevalence in short-legged, long-backed breeds such as Dachshunds and French Bulldogs (1–3). The core issue of this disease lies in the degenerative changes or injuries of the intervertebral disc, leading to the extrusion of the nucleus pulposus or annulus fibrosus, which compresses the spinal cord or surrounding nerves. This results in pain, neurological disorder, and even paralysis (1, 3–7). Currently, MRI is the golden standard for diagnosing IVDD.

The treatment of canine intervertebral disc disease can be categorized into conservative and surgical approaches. The choice of treatment largely depends on the severity of the neurological signs. According to the study by Crawford et al., conservative treatment (a combination of analgesic medication and restricted exercise) had been found suitable for dogs with mild disc extrusion and less severe spinal cord compression, achieving a success rate of 29% (8). Surgical treatment is indicated for dogs with more severe disc damage. Statistics had shown that open surgical procedures (a hemilaminectomy with anulectomy or with a partial discectomy) had achieved a success rate of 71% (8).

Currently, common techniques for thoracolumbar spinal cord decompression include hemilaminectomy and mini-hemilaminectomy. Those techniques are typically performed using an open approach (the muscle are disinserted from the bone extensively) allowing the surgeon to visualize the anatomical landmarks. Minimally invasive techniques have emerged in recent years in veterinary neurosurgery. This shift has been possible via the use of magnification and illumination technique. Endoscopic spinal surgery is gaining a lot of traction in human minimally invasive spine surgery (MISS) but little is known about the technique in veterinary medicine. Compared to traditional open surgeries, endoscopic surgeries offer smaller incisions, less tissue trauma, improved visibility, and a lower complication rates (9). Reports on the use of endoscopy for treating intervertebral disc disease in veterinary medicine had been limited (10–16). Moon et al. had demonstrated the feasibility of percutaneous endoscopic thoracolumbar mini-hemilaminectomy via a uniportal approach in small dogs. Although their study had simulated intervertebral disc extrusion using an injected barium and agarose mixture, the findings had indicated that this surgical technique was effective in alleviating spinal compression. It had provided a good view during the operation, required only a small skin incision (<1 cm), and had a relatively short operation time (58 min) (11). Hwang et al. had performed percutaneous endoscopic thoracolumbar pediculectomy on five healthy dogs at the thoracic spine and five healthy dogs at the lumbar spine. Postoperatively, only one dog had exhibited a transient, slight ipsilateral hind limb weakness, which had resolved within 4 days. The gait and neurological examinations of the other dogs had been normal. Two dogs had showed high signal intensities within the spinal cord on T2-weighted (T2W) magnetic resonance imaging (MRI), suspected to have been spinal cord edema or gliosis, which had improved after 4 weeks. These findings suggest that percutaneous endoscopic pediculectomy might have been an effective surgical method for decompression and removal of disc material in canine intervertebral disc disease (13).

Currently, there have been no reports on the use of the “single-incision double-channel mini-open” technique for treating thoracolumbar intervertebral disc disease in dogs. The uniqueness of this technique lies in achieving double-channel operation through a single incision, which not only retains the advantages of traditional endoscopic surgery, such as less intraoperative injury and rapid recovery, but also significantly enhances operational flexibility and visibility. This represents an important innovation in the treatment of intervertebral disc disease in dogs.

This study aims to describe a minimally invasive surgical technique for endoscope-assisted single-incision double-channel mini-open hemilaminectomy in the treatment of acute thoracolumbar intervertebral disc disease in dogs. The study reports on 11 cases treated with this technique and evaluates its efficacy in removing herniated disc material and achieving spinal cord decompression. To assess potential postoperative complications, neurological examinations, MRI, and computed tomography (CT) scans were conducted, and the clinical outcomes of the cases were followed to evaluate the safety of the technique.

## 2 Materials and methods

### 2.1 Case information and preoperative examination

This study included 11 dogs diagnosed with thoracolumbar intervertebral disc disease. All cases were of acute onset. Information such as breed, age, weight, and sex was recorded for all cases in Table 1. Physical examinations and neurological assessments were performed on each animal. Neurological assessments for all cases were conducted by a senior surgeon, focusing primarily on evaluating mental status, gait analysis, spinal reflexes, palpation, and pain perception. The neurological grading of the case follows the published scoring system (17). The grading system was as follows: grade 0, normal gait; grade 1, thoracolumbar pain with no neurological deficits; grade 2, ambulatory paraparesis; grade 3, non- ambulatory paraparesis; grade 4, paraplegia with intact deep pain perception in at least one limb; and grade 5, paraplegia with loss of deep pain perception. “Ambulation” was defined as the ability to walk 10 consecutive steps without support as previously reported (18). Complete blood count and blood biochemical analyze were performed before surgery; MRI (1.5 Tesla, uMR580, United Imaging Medical Technology Co., LTD, Shanghai, China) and CT (uCT 768, United Imaging Medical Technology Co., LTD, Shanghai, China) were used to diagnose thoracolumbar intervertebral disc disease in all cases and to identify the location of the affected intervertebral discs.

**Table 1:** Basic characteristics of dogs undergoing mini-hemilaminectomy assisted by endoscopy, including breed, sex, age, weight, neurological grading, and the location of the affected intervertebral disc.

Case	Breed	Sex	Age	Weight	Neurological grading*	Lesion location
1	French Bulldog	Male	4Y	13.2 kg	2	T13-L1
2	Pomeranian	Female	3Y	4.3 kg	3	T13-L1
3	Shiba Inu	Male	6Y	18 kg	3	L2-L3
4	Corgi	Female	5Y	16 kg	4	L2-L3
5	Chinese rural dog	Male	5Y	6.8 kg	4	T12-T13
6	Bichon Frise	Male	7Y	6.1 kg	5	L1-L2
7	Poodle	Male	7Y	9.6 kg	4	T12-T13
8	German Shepherd	Male	7Y	46 kg	2	L1-L2
9	Corgi	Male	9Y	14 kg	3	T13-L1
10	Corgi	Male	7Y	15 kg	4	T12-T13
11	Corgi	Male	5Y	13.7 kg	4	L1-L2

Y, years old; kg, kilogram.

* The grading system was as follows: grade 0, normal gait; grade 1, thoracolumbar pain with no neurological deficits; grade 2, ambulatory paraparesis; grade 3, non- ambulatory paraparesis; grade 4, paraplegia with intact deep pain perception in at least one limb; and grade 5, paraplegia with loss of deep pain perception.

Each dog was preoperatively administered butorphanol intravenously (0.2 mg/kg). Anesthesia was induced with intravenous propofol (6 mg/kg, administered to effect), followed by maintenance with inhalation of 1.5% isoflurane in oxygen via endotracheal intubation. During the surgery, dexmedetomidine was infused intravenously at a rate of 1 μg/kg/h for intraoperative analgesia, and lactated Ringer’s solution was administered at 5 mL/kg/h. Additionally, ceftriaxone (25 mg/kg) was used intraoperatively, with doses repeated every 90 min. The dogs were placed in a sternal position, and the surgical site was aseptically prepared.

### 2.2 Surgical procedure

**Figure 1:**
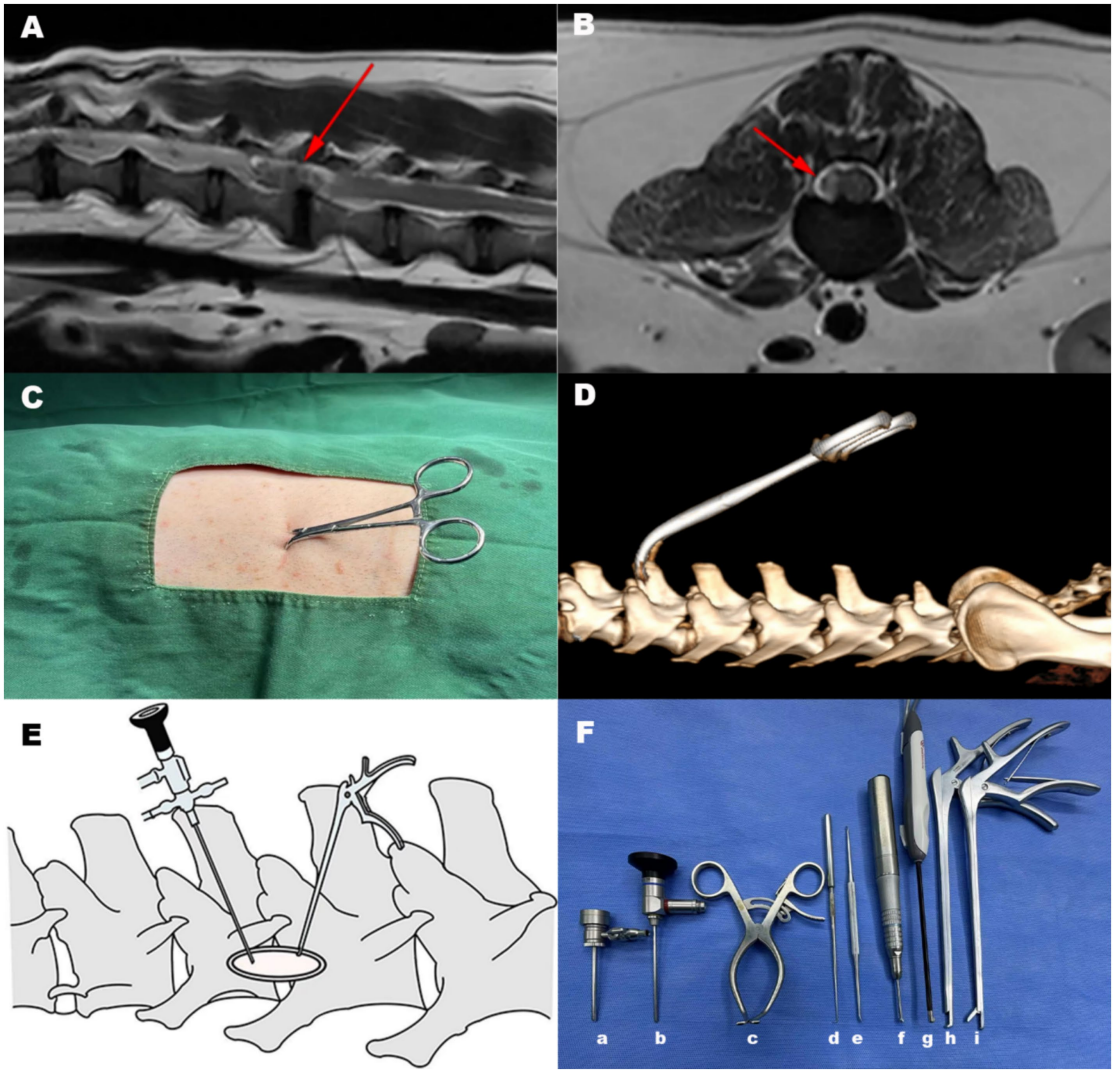
Surgical positioning and approach. **(A)** Locating the intervertebral disc for surgery using the MRI T2-weighted mid sagittal image. **(B)** Locating the intervertebral disc for surgery using the MRI T2-weighted transverse image. The arrow indicated the location showing the intervertebral disc lesion. **(C)** Palpation was used to secure the spinous process of the cranial vertebra at the lesion site with a sterile drape forceps. **(D)** After clamping the spinous process, the clamp position was confirmed via CT scan. **(E)** A modified unilateral biportal endoscopic surgical approach was adopted. A 2 cm incision is made just below the adjacent articular process to the damaged intervertebral disc, with the center of the incision aligned with the articular process. An endoscope with a sheath is inserted on one side of the incision, and surgical instruments are introduced on the other side of the incision. **(F)** Surgical instruments used in Endoscope-assisted single-incision double-channel mini-open hemilaminectomy, (a) Endoscope sheath (b) Endoscope (c) Gelpi retractor (d) Nerve hook (e) Periosteal elevator (f) Electric drill (g) Plasma cutter head (h) Rongeur (i) Nucleus pulposus forceps.

The lesion site was identified preoperatively using MRI (Figures 1A,B). After shaving and aseptically preparing the surgical area, the dog was transferred to the CT table. The spinous process of the vertebra on the cranial side of the affected disc was palpated and clamped with sterile drape forceps. A CT scan was performed to confirm the correct clamping position (Figures 1C,D). The dog was then transferred to the surgical table, and the surgical site was re-disinfected. Sterile drapes were applied, and a thin sterile film was placed over the drapes to prevent spillage of saline irrigation during the surgery from soaking the animal. Based on the position of the drape forceps, the adjacent articular processes near the affected intervertebral disc were palpated. A 2 cm incision was made just below the articular process, with the incision center aligned with the articular process. During surgery, a vital signs monitor (ePM 12 M Vet, Shenzhen Mindray Bio-Medical Electronics Co., Ltd., Shenzhen, China) is used to track the heart rate, oxygen saturation, blood pressure, and end-tidal carbon dioxide levels.

**Figure 2:**
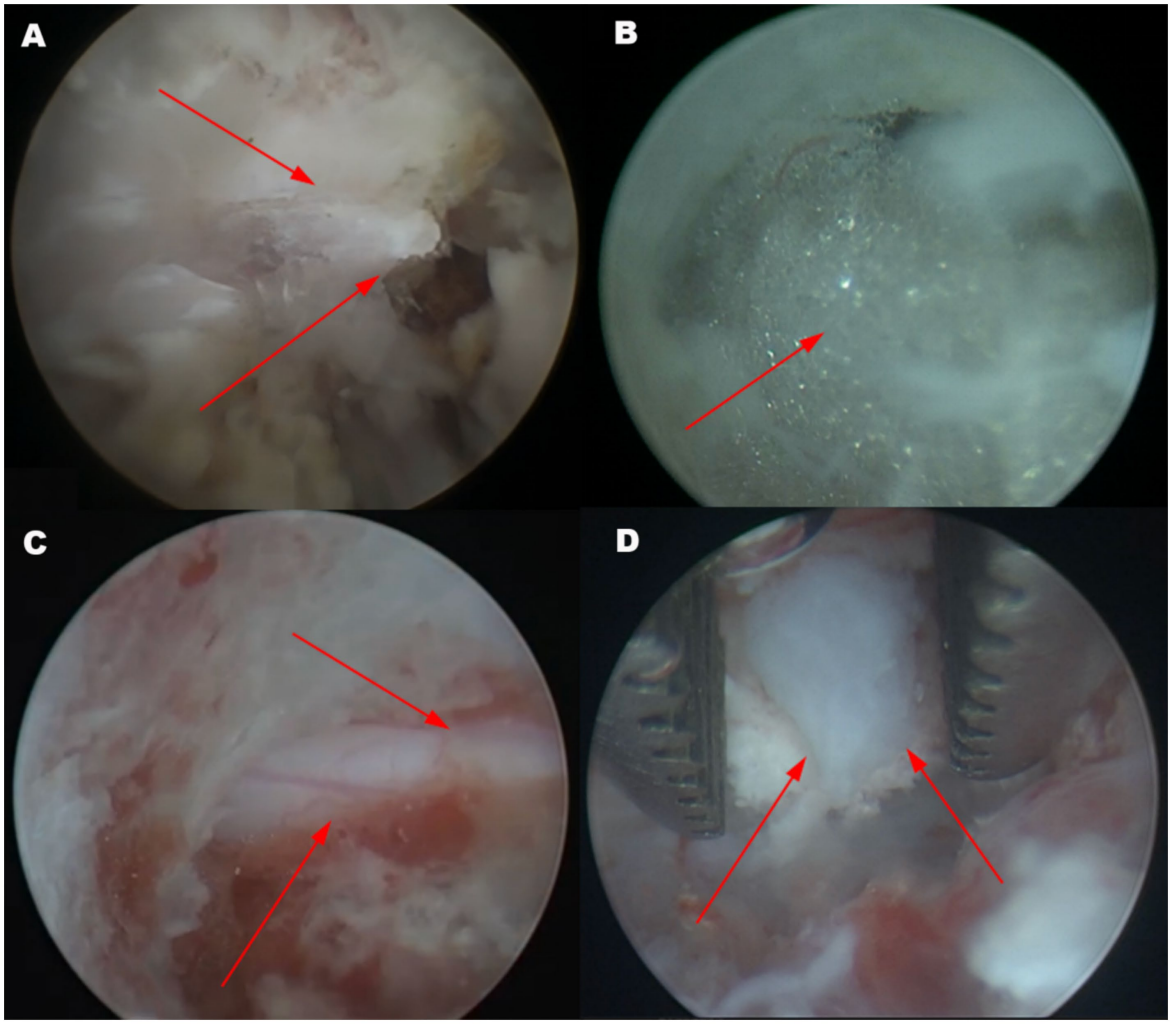
Endoscopic view of the surgical sites. **(A)** The tendinous attachment points of the accessory process can serve as a navigationa marker. The accessory process can be clearly observed (arrow). **(B)** Fat tissue inside the spinal canal becomes visible after drilling through the lamina with a bur (arrow). **(C)** Upon enlarging the approach, the spinal nerve root can be observed (arrow). **(D)** The compressed disc tissue is removed using a nucleus pulposus forceps. The arrow pointed to the herniated intervertebral disc.

Using coblation system (ASC4250-01, ArthroCare Corporation, Texas, USA) and a periosteal elevator (3/3 mm, 155 mm; Puenhua, Jiangsu, China), the epaxial musculature below the articular process was separated to expose the lamina, and used a Gelpi retractor to secure the wound opening. The coblation system minimized bleeding and caused minimal thermal damage to tissues, promoting faster wound healing. During dissection, care was taken to note the tendinous attachment points of the accessory process, which served as navigation marker (Figure 2A). After sufficient exposure, an endoscope (30°viewing angle, diameter 2.4 mm, length 73 mm; Hangzhou VetLuc Co., Ltd., Zhejiang, China) with a sheath (diameter 2.9 mm, length 58 mm; Hangzhou VetLuc Co., Ltd., Zhejiang, China) was inserted on one side of the incision, surgical instruments were introduced on the other side of the incision (Figure 1E). The positions of the endoscope and surgical instrument can be interchanged depending on the specific situation. Using an infusion pressure bag (500 mL, Vega (China) Instrument Co., Ltd., Zhejiang, China), normal saline was continuously infused into the surgical site through the endoscopic channel. The pressure was set to 30 mmHg, with a flow rate of 0.4 L/min. A right-angle suction tip was placed at the edge of the wound, and a suction device (7A-23D, Yuwell Medical Equipment Co., Ltd., Jiangsu, China) was used to remove excess fluid from the wound. Under endoscopic visualization, the accessory process was breaked using the periosteal elevator and further ground down with a electric drill (TPS MicroDrill, Stryker, Michigan, USA). After grinding away the outer cortical bone and cancellous bone, advancing inward with the bur until the lamina was penetrated and epidural fat was visible (Figure 2B). A laminectomy rongeur (PC3260, 1 mm/210 mm, Shanghai Medical Instruments Co., Ltd., Shanghai, China) was used to enlarge the view. Bone debris was flushed out with saline irrigation. At this stage, the spinal nerve root could be observed (Figure 2C). Using a nerve hook (600,011, Byers, Jiangsu, China) and a nucleus pulposus forcep (2 mm/220 mm, Hongda Medical Equipment Co., Ltd., Jiangsu, China), the compressive intervertebral disc material was removed under endoscopic visualization (Figure 2D). This process continued until spinal cord decompression was achieved and no further disc material could be removed. The nerve hook was passed below the spinal cord and through the entire intervertebral disc space to confirm the absence of residual disc extrusion. The surgical site was flushed with saline, and the endoscope and instruments were removed. The surgical site was closed routinely. Bleeding during surgery was one of the challenges that needed to be addressed. We typically used coblation system, compression, or hemostatic sponges for hemostasis. The main surgical instruments were shown (Figure 1F).

### 2.3 Postoperative care

MRI or CT were performed immediately after surgery to evaluate the decompression of the spinal cord. If preoperative CT imaging clearly showed the lesion, CT was preferred. Then the patients were hospitalized for 1 week for closely monitoring. During hospitalization, the condition of animals was closely monitored to prevent recurrence of spinal cord compression and complications. Additionally, postoperative monitoring helps assess the recovery of neurological function. Postoperatively, nonsteroidal anti-inflammatory drugs (NSAIDs) and butorphanol were administered for pain control. Sutures were removed 10–14 days after the surgery. From the third postoperative day, rehabilitation and laser therapy were used for the patients until they got discharged.

## 3 Results

### 3.1 Surgical technique evaluation

All cases were successfully treated. During the procedure, the combination of an endoscope and an irrigation system was used to flush out debris from drilling, ensuring a clear surgical field. The muscle attachment point of the accessory process served as a navigation marker, providing critical reference for precise operations. Ultimately, the herniated disc material was precisely removed under endoscopic visualization, effectively relieving spinal cord compression. In all dogs, the affected intervertebral discs were clearly exposed, herniated disc material was successfully removed, and the nerve roots and blood vessels were systematically identified and preserved. As mentioned earlier, bleeding was one of the biggest challenges during the surgery. Sometimes, it was difficult to control and affected the surgical procedure. In such cases, we paused the irrigation and the surgery to focus on hemostasis first. The surgery duration was approximately 80–90 min.

### 3.2 Case outcome

All dogs received laser therapy and rehabilitation treatment postoperatively to promote healing and restore motor function, with no significant complications observed. By the fifth postoperative day, all dogs exhibited normal gait and neurological examination results (Olby score of 14) (19).

### 3.3 CT and MRI evaluations

All affected dogs exhibited varying degrees of neurological disorders before surgery, with neurological grades ranging from 2 to 5. Preoperative imaging revealed different degrees of nucleus pulposus extrusion and intervertebral disc degeneration in all 11 dogs, with the extruded nucleus pulposus spanning 1 to 2 vertebral segments and the area of spinal cord compression ranging from 30 to 50%. No significant spinal cord edema or hemorrhage was observed.

**Figure 3:**
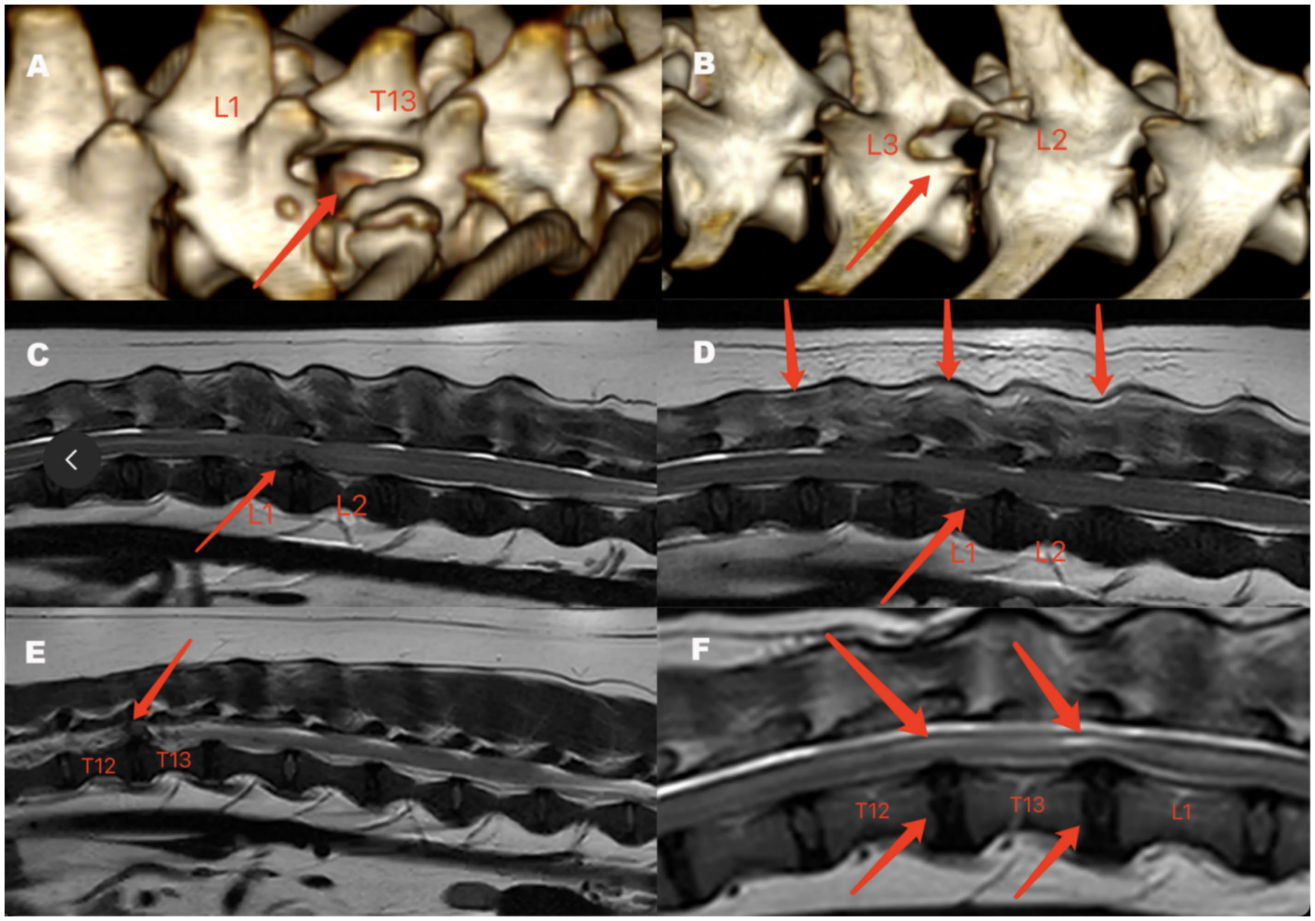
Postoperative 3D-reconstruction image of Case 1 **(A)** and Case 3 **(B)**, preoperative and postoperative MRI findings of Case 4 **(C,D)** and Case 11 **(E,F)**. **(A)** Lateral view of the 3D reconstructed image of Case 1, with surgery performed on the right side (arrow). **(B)** Lateral view of the 3D reconstruction of Case 3, with the surgery performed on the left side (arrow). **(C)** Preoperative MRI of Case 4 in the T2-weighted mid sagittal image shows low to moderate signal material within the spinal canal compressing the spinal cord, with no significant abnormality in the spinal cord signal. The arrow indicated the affected intervertebral disc. **(D)** Postoperative MRI of Case 4 on day 3 in the T2-weighted mid sagittal image shows the disappearance of the abnormal signal material in the spinal canal, no significant abnormality in the spinal cord signal (lower arrow), and a mixed moderate-to-high signal observed in the muscles surrounding the foraminal endoscopic surgery site (upper arrow). **(E)** Preoperative MRI of Case 11 in the T2-weighted mid sagittal image shows low to moderate signal material within the spinal canal compressing the spinal cord. The arrow indicated the affected intervertebral disc. **(F)** Postoperative MRI of Case 11 at 16 months in the T2-weighted mid sagittal image shows localized high signal intensity in the spinal cord at the surgical site segment (upper arrow), mild disc protrusion, and no signs of spinal cord compression (lower arrow).

The 3D reconstructed image showed that after performing an endoscope-assisted single-incision double-channel mini-open hemilaminectomy, the surgical window was clearly visible (Figures 3A,B).

Due to the concern on anesthesia for postoperative MRI and CT evaluations, and in accordance with the owners’ preferences, we collected postoperative MRI data for only two cases. In case 4, during the initial MRI scan, on the T2W (sagittal) image, a material with low to moderate signal was observed within the vertebral canal, compressing the spinal cord, while the signal of the spinal cord parenchyma showed no significant abnormalities (Figure 3C). On the third postoperative day, the loss of abnormal signal material in the vertebral cavity can be seen below the T2W (sagittal) image, with no significant abnormality in the spinal cord signal, while the muscles around the surgical site (accessory process) showed moderate to high signal intensity (Figure 3D). In case 11, during the initial MRI scan, on the T2W (sagittal) image, a material with low to moderate signal was observed in the vertebral canal, compressing the spinal cord (Figure 3E). In the follow-up MRI 16 months after surgery, on the T2W (sagittal) image, the surgical site segment of the spinal cord showed a localized high signal intensity, with mild disc protrusion, but no signs of spinal cord compression (Figure 3F).

## 4 Discussion

Minimally invasive treatment of intervertebral disc disease is generally considered challenging, primarily due to the complex anatomical structure of the spine, the presence of multiple muscle tissues, nerves, and blood vessels nearby, and the proximity of the surgical site to the spinal cord. This study presents a minimally invasive neurosurgical technique for treating canine intervertebral disc disease. The study included 11 dogs treated with this surgical technique, all of which showed favorable imaging results postoperatively. Physical and neurological examinations of all dogs were normal.

The magnification and illumination functions of the endoscope can be achieved through the lens and cold light source or LED light source, enabling the magnified observation of small structures. This magnification and illumination capability helps veterinarians see fine details of the surgical site more clearly in darker or narrower surgical areas, which is particularly useful in surgeries involving the nervous system.

In human medicine, unilateral biportal endoscopy is a novel minimally invasive technique for treating thoracolumbar intervertebral disc herniation. The surgical approach involves making two incisions approximately 1 cm lateral to the dorsal midline, with saline infusion entering through one port and suction exiting through the other (20). In our preliminary stage of exploring the surgical approach, this technique was also tested on canine cadavers. The results showed that due to the looser skin in dogs and the larger space between the skin and muscle tissue, the infused saline would accumulate subcutaneously. Therefore, through modification and optimization, the approach was adjusted to use a single incision, with the suction device placed at the incision edge to effectively drain the fluid.

In other reports, the surgical site had been located and the working channel had been established under fluoroscopic guidance, using a Kirschner wire (K-wire) to locate the damaged intervertebral disc. After determining the position, a K-wire or spinal needle had been inserted and advanced medially to the pedicle until bony resistance had been met. Then, a series of gradually increasing dilators had been placed over the K-wire, and finally, a tubular retractor had been used to establish the working channel. Some studies had also employ a articulated arm to hold the retractor for the working channel (11, 13, 14). The positioning in this study was achieved using CT imaging, with surgical localization assisted by palpation and visual inspection, eliminating the need for fluoroscopy and simplifying the procedure.

In the reported surgical techniques, most had used a single-incision approach, where both the endoscope and other surgical instruments had been placed within the tubular retractor, with the surgical incision being approximately 1 cm in length (11, 13). In contrast, this study used a single-incision, double-channel approach for surgery. Although the surgical incision was relatively longer (about 2 cm), the double-channel surgical path provided more operating space. Tanaka et al. reported a lateral muscle-separating approach similar to the surgical approach in this study. The extent of the skin incision was from the posterior border of the articular process to the rostral vertebra and from the anterior border of the articular process to the caudal vertebra, with an average incision length of 2.7 cm. The incision length was correlated with the size of the dog. In this study, the surgical incision was made as a 2 cm cut below the articular process, regardless of the dog’s size (21). In our research, a 2 cm incision was sufficient to perform the surgical procedure. Spinal surgery is a technically demanding and challenging procedure and veterinarians must perform the surgery carefully and precisely, as even a slight error can damage nerves and lead to severe consequences. The single-incision, double-channel surgical approach was easier to perform, reducing the likelihood of iatrogenic injuries and improving the safety and stability of the surgery. Additionally, the surgical equipment and instruments used in this study were fewer and relatively easy to obtain.

Our study also has some limitations. For large dogs and obese dogs, where it was difficult to palpate the vertebral processes, drape forceps were not able to grasp the spinous processes. Additionally, the approximate surgical time was recorded in this study, which was around 80–90 min, longer compared to open hemilaminectomy (22). It should be noted that this surgical technique relies on endoscopic assistance, making the surgical costs higher than open surgeries that only require conventional spinal surgical instruments. Another limitation was that this surgical technique was suitable for cases where the prolapsed nucleus pulposus was located ventral to the spinal cord. For lateral or dorsolateral spinal extrusion or intradural extrusion, it might have been necessary to extend the laminectomy dorsally.

In our study, we reported a surgical technique using minimally invasive methods to treat canine intervertebral disc disease, which allowed for spinal cord decompression and removal of herniated disc material. Postoperative results indicated that the affected dogs recovered well without significant complications. Compared to traditional open surgery, this method offered advantages such as precise localization, reduced iatrogenic injury, and improved visibility. The use of a dynamic irrigation system during the procedure ensured a clear surgical field. Compared to the single-port approach, the double-channel design provided greater operating space, enhancing the safety and operability of the surgery. However, the sample size in this study was small, and there was a lack of long-term follow-up data to assess long-term efficacy. Further studies are needed to evaluate the application of this method in dogs with thoracolumbar intervertebral disc disease.

## Data Availability

The raw data supporting the conclusions of this article will be made available by the authors, without undue reservation.
