# Supplementary material for: Case Report: Endoscope-assisted single-incision double-channel mini-open hemilaminectomy for the treatment of acute thoracolumbar intervertebral disc disease in 11 dogs
Source: Front Vet Sci. 2025 Apr 9;12:1543611. doi: 10.3389/fvets.2025.1543611 (PMC12016880; doi:10.3389/fvets.2025.1543611)
Supplement: Supplementary file 1 [file Table_1.docx]

**Supplementary Table 1.** Follow-up records of 11 cases of acute thoracolumbar intervertebral disc disease treated with endoscope-assisted single-incision double-channel mini-open hemilaminectomy.

| Case | Preoperative  Neurological grading* | postoperative short-term (1 week)  Neurological grading* | short-term/long-term follow-up results |
| --- | --- | --- | --- |
| 1 | 2 | 2 | Two weeks postoperatively, animals returned to normal walking |
| 2 | 3 | 3 | Three weeks after surgery, animals returned to normal walking |
| 3 | 3 | 2 | Two weeks postoperatively, animals returned to normal walking |
| 4 | 4 | 1 | Two weeks postoperatively, animals returned to normal walking |
| 5 | 4 | 3 | Four weeks after surgery, animals returned to normal walking |
| 6 | 5 | 4 | Five weeks after surgery, animals returned to normal walking |
| 7 | 4 | 2 | Two weeks postoperatively, animals returned to normal walking |
| 8 | 2 | 1 | One week postoperatively, animals returned to normal walking |
| 9 | 3 | 2 | Two weeks postoperatively, animals returned to normal walking |
| 10 | 4 | 2 | Three weeks after surgery, animals returned to normal walking |
| 11 | 4 | 3 | Two weeks postoperatively, animals returned to normal walking |

** The grading system was as follows: grade 0, normal gait; grade 1, thoracolumbar pain with no neurological deficits; grade 2, ambulatory paraparesis; grade 3, non- ambulatory paraparesis; grade 4, paraplegia with intact deep pain perception in at least one limb; and grade 5, paraplegia with loss of deep pain perception.*
